# Editorial

**Published:** 1870-10

**Authors:** 


### Editorial.

Medical Colleges in Chicago.—The next annual course of instruction in the Chicago Medical College and Medical Department of the Northwestern University will commence on Monday, October 3, 1870.

The Introductory Lecture will be given in the evening, by Prof. H. A. Johnson. As it will be the formal opening of the new college building, it is expected that the President and Faculty of the University will be present and participate in the exercises, thus making the occasion one of more than ordinary interest. The College is on the corner of Prairie Avenue and Twenty-sixth Street, in immediate connection with the Mercy Hospital and Dispensary.

The Rush Medical College commences on Wednesday, the 28th of September.

The Woman's Hospital Medical College commences on Tuesday, October 4th.

Chicago now has a population of about 300,000, and her colleges and hospitals afford as good facilities for medical instruction *in every department,* as can be found in any city in the United States.

New Work.—We see by some of our exchanges that Prof. Byford's work on the "Theory and Practice of Obstetrics," has been published by Wm. Wood & Co., of New York. It is represented as an elegant volume of 450 pages, well-illustrated with cuts, and well-adapted for the use of students and practitioners. It is less voluminous than most of the standard works on Midwifery, and yet sufficiently full in direct practical details.

We shall notice it more at length when we receive a copy of the work.

---

British Medical Association.—The Annual Meeting of this Association commenced in Newcastle-on-Tyne, August 9, 1870. Through the kindness of our friend, Dr. S. J. Jones, who was present at the meeting, we have been furnished with full reports of each day's proceedings, and abstracts of the various papers presented to the Sections.

The sessions were continued four days. The papers prepared for the Sections numbered more than fifty; some of which were highly important.

Mr. Furneaux Jordan, presented a paper on the "Treatment of Enlarged Lymphatic Glands," in which he advocated strongly the maintainence of counter-irritation, not over, but in the vicinity of the gland affected. For instance, in enlarged glands of the neck, he makes decided counter-irritation with iodine over the back of the neck, extending it to a line a little below the diseased glands. He also recommends pressure with *shot-bags* on the tumors, as much as the patient can bear in a recumbent position, for a little while each day. By these means he claims unusual success in the treatment of this class of troublesome ailments.

M. Teevan, read a paper on "Spermatorrhœa," in which he made an important distinction between true Spermatorrhœa, and involuntary nocturnal emissions. The former was characterized by the unconscious passage of the Spermatozoa in the urine, and was almost always accompanied by a loss of sexual desire, and resulted from habits of debauchery, indigestion, and local irritants. The pathological condition is one of loss of sensibility, and consequent relaxation of the seminal ducts, and is most likely to be relieved by good hygienic regulations, chalybiate tonics, strychnine, and electricity. Sometimes the application of nitrate of silver to the orifices of the seminal ducts, is useful; but bromide of potassa and other nervous sedatives are worse than useless. On the other hand, the usual nocturnal seminal emissions depend on a morbid excitability of the sexual organs, with excessive sexual desire, in most cases.

Such cases are much more likely to be relieved by free, habitual, muscular exercise, plain diet, and pretty full doses of the bromide of potassa taken at bedtime. In the first variety, continence or entire abstinence from sexual intercourse is regarded by M. Teevan as essential to recovery. Our space in the present issue will not permit us to notice more of the interesting papers read in the several sections.

The general meetings of the Association, which were held a part of each day, were occupied mostly with the subject of medical education and examinations.

The British Medical Association appears to be occupied in the same work for which our own national organization was instituted. The great central evil of the system of medical education in Great Britain, and the remedy, are very clearly stated by the President, Dr. Charlton, in his opening address. He says, "Although the deficiencies of medical education, and the condition of the medical practitioner, had long before been discussed in our journals, and been set forth in language sharp and incisive as that of Junius, still it is only within the last two decades, that the whole profession, tired of calling upon Jupiter, has unanimously put shoulder to the wheel, and moved with one accord to remedy our wrongs. United by its able journal, united still more by the cordiality engendered at these annual meetings, the British Medical Association has pushed forward in the path of medical education, and the general regulation of the profession; and, now that final struggle for a good standard of medical education is finally before us. Hitherto the paramount obstacle to progress has been the multiplicity of sources from whence licenses to practice medicine have been derived. No uniform standard of education could be kept up amid these jarring interests. The well-meant endeavors of the Medical Council, by sending out examiners to visit the different universities and corporations, was no guarantee for permanent improvement; these bodies were ever competing for licentiates, and were exposed to the deadly temptation of lowering their standards of education to attract more graduates. Against this crying evil, there was but one remedy—a single gate by which all should enter the profession; a single but searching examination for the license to practice." The evil of having a score of institutions, each granting diplomas or licenses to practice, and each pecuniarily interested in increasing the number of its graduates, appears to be clearly appreciated by a large majority of our British brethren. And we are glad to see that their Association contends firmly and vigorously for the only efficient remedy; namely, the formation of a single, independent board of examiners. Their position, with any argument brought to sustain it, applies with even greater force to the condition of things in our own country. So long as we have thirty or forty colleges or schools, issuing diplomas which are recognized as licenses to practice, so long will the competition for larger classes, entirely prevent the adoption and maintainence of a proper standard of attainments, either preliminary or medical.

We were fully satisfied with the correctness of this position nearly thirty years since, when discussing the subject in the Medical Society of the State of New York. And all our observations in the American Medical Association, and in the Conventions of Delegates from Medical Colleges, have only served to strengthen our convictions.

All admit that we ought to have a higher standard of preliminary requirements before young men are permitted to enter upon the study of medicine. All equally admit that the method of pursuing medical studies in the schools (with one or two exceptions) is destitute of any consecutive order, too limited in time, and in many instances too limited in facilities for clinical observation. Yet so potent and universal is the idea that a large part of the students of medicine will attend such schools as will give them a diploma in the shortest time, and with the least attention to either literary or professional qualifications, that the schools of no one city dare to add a month to the length of their lecture term, or exact any adequate preliminary qualifications, &c., lest the schools of neighboring cities should reap advantages by keeping their time and requirements less. It is thus that the union of the teaching and licensing in the same institution, operates as a constant and almost insuperable barrier to any noted improvement in the education of the profession. The sooner the profession, both in England and America, devise a method by which admission into the ranks of the profession shall depend on a full examination by independent boards, leaving the schools to compete with each other solely on the basis of their merits as teaching bodies, the sooner will the great evils and defects in medical education be remedied.

Opening of the Medical Colleges.—An unexpected delay of a few days in the issuing of this number of the *Examiner*, enables us to add a few words in regard to the opening of the Medical Colleges in this city. The Rush Medical College opened its session on the evening of the 28th of September, with an introductory from Prof. Morse on the Mind. The Lecture-room was well filled, and the number of students in attendance nearly the same as at the opening of former sessions.

The Chicago Medical College opened its Twelfth Annual Course of Instruction in its new building, on the corner of Prairie Avenue and Twenty-Sixth Street, on the evening of October 3d. The spacious Lecture-room was well filled with an audience of gentlemen and ladies, in addition to the students. The introductory lecture was delivered by Prof. H. A. Johnson, and as the completion of the new building, with the permanent connection of the institution with the Mercy Hospital on the one hand, and the Northwestern University on the other, marked an era in the progress of the College, the lecture was largely historical. It was listened to with marked attention, and we hope to give it to our readers in full in the next number of the *Examiner*. The number of matriculants in the College, at the opening, was nearly double the number that had been registered at the opening last year.

The Woman's Hospital Medical College opened its first College Term on the evening of the 4th inst. The exercises were held in the Clark Street Methodist Church. The introductory lecture was delivered by Prof. W. H. Byford, and was listened to by a large audience. What the prospects are for a class we do not know.

---

Strychnine as an Antidote for Chloral Poisoning.—At a meeting of the Berlin Medical Society, in November, 1869, Dr. Liebreich communicated the result of his experiments in search of an antidote for chloral poisoning.

His investigations have not been directed to the discovery of a neutralizing agent for chloral while it remains in the alimentary canal, but after it had entered the circulation. The primary action of chloral being upon the brain and spinal cord, and its secondary upon the heart, he had sought for a remedy which had its immediate effect upon the latter organ.

It occurred to him that strychnine might meet this indication, which increased the force of the systole and lessened that of the diastole, the reverse effect of chloral. Experiments upon animals had confirmed its antidotal properties; its efficacy, however, being restricted to the profounder states of chloral asphyxia.

If merely hypnotic doses of chloral be given to an animal, the administration of strychnine will induce the usual tetanic phenomena. This does not occur where the dose of chloral has been sufficieut to affect the function of the spinal cord.

The fact was shown by the following experiments: A fatal dose of chloral was given to a rabbit, and death shortly ensued. The same quantity was given to a second, but as the respiration began to flag, a fatal dose of strychnine was injected (hypodermically). It soon roused from stupor, and perfectly recovered. Two days afterward the same quantity of strychnine was again given, and death occurred in fifteen minutes. He therefore advises its use where the life of the individual is threatened from over-doses of chloral, at the same time employing the ordinary irritants and excitants.

During the discussion which followed the reading of the report, Dr. Von Langenbeck suggested that it would be difficult to determine the quantity of strychnine to be used subcutaneously in asphyxiated states from either chloroform or chloral.

Dr. L., from his experiments on animals, estimates the dose at about 10 milligr. for adult individuals.—*Memorabile Heilbronn, March, 1870.*

---

REPRODUCTIONS OF LIMBS.—M. Philepeaux has proved for fish what he already demonstrated in the case of newts, *viz.*, that when the limb is removed below the scapula or ilium, it is reproduced. But when the scapula or ilium is removed, no reproduction takes place.—*Monthly Microscopical Journal.*

### SOME CORRECTIONS.

ZANESVILLE, OHIO, *September 6, 1870.*

PROF. DAVIS:—For the general accuracy of statement in your review of my fasciculus on "The Bromides," please accept my thanks; but you have fallen, inadvertently, no doubt, into several errors, which please allow me to correct.

You state that I am carrying out the train of thought in my published papers prominently enunciated by Prof. Huxley. This is a mistake, both of fact and chronology. My classification of therapeutic agents, based on the repair and waste of the tissues was first announced in a critical study of "Alcohol, as Viewed by the Eye of Science;" communicated, as a valedictory, to the Muskingum County Medical Society, May 1, 1867; afterwards published in *The Medical Archives*, St. Louis, June, 1869. The valedictory for 1868, on "How do Medicines Produce their Effects?" was a further elaboration of life from a physical basis, published in the St. Louis *Medical Reporter*, for December 15, 1868. That on "The Bromides," was read as a valedictory, May 5, 1869, and published, as you state, in the New York *Medical Journal*, for July, 1870.

Prof. Huxley's lecture on "Protoplasm," or "Physical Basis of Life," was delivered in Edinburgh, November 18, 1868; and was first published in London, in *The Fortnightly Review*, for February, 1869. Near the close of April, 1869, and just before the memoir on "The Bromides" was delivered, I saw a notice of Prof. H.'s lecture in *The Book Buyer*, in Mr. Welford's London letter. Though it had already appeared in *Every Saturday*, in Boston, I did not know it for several months after. An abridgment of it first met my eye in *Appleton's Journal*, May 15, 1869, ten days after the paper had been read to the Society. The words, or sentence, physical basis of life, as used in it, are in a wholly different sense from that in which Prof. H. uses them in his lecture.

I have no desire to detract anything from the well-earned laurels of Prof. H.; but truth compels me to say I derived

* *Chicago Medical Examiner*, September, 1870, p. 584.

no aid from any of his published papers, and I never saw or corresponded with him; and, if I understand him, he regards what we call "life," as a property of matter, which is certainly not the teaching of any of my published papers, as the diagram in that on "The Bromides" demonstrates.

Placing me, therefore, as a follower of Prof. Huxley is a mistake of fact, and, as I here show you, the identical views contained in my paper on "The Bromides" were published in this country nearly a year before the delivery of his lecture on "Protoplasm," in Edinburgh.

Again, you state that my conclusion is that the bromides are applicable "to the treatment of such cases only as are accompanied by the retention of '*surplus material.*'" My statement was the retention of *effete matter*, between which and a mere surplus, there is just the difference between life and death, no more and no less.

A future exact science of life and therapeutics must grow out of what we call physical science, and no basis of therapeutics can be more simple or exact than one based on the supply and waste of the tissues, as it is in them, force is, so to speak, stored up. Nor can any basis of physiology be more exact and simple than that which bases normal function on normal forms, and molecular changes in the material of the forms of the tissues; nor of pathology, or loss of form, or increase or decrease of the velocity of molecular changes in the material of forms; seeing that these are the absolutely naked scientific conditions of life.

Whatever may be my merits or demerits in this connection, they must rest on the practical application of physical and chemical law in actual clinical medicine, and from several year's experience, I find it so much simpler, as well as so much more certain, it must be nearer right than that now in use by the profession.

I hope, before the close of the year, to submit you a report of a clinical case, managed in accordance with life from a physical basis, and therapeutics from supply or repair and waste of the tissues, for insertion in *The Examiner*. Very truly yours,

Z. C. McELROY.

THE STETHO-SPHYGMOGRAPH. BY T. HAWKSLEY, M.D., M.R.C.P.—At a meeting of the Medical Society of London, on Nov. 8th, Dr. Hawksley read a paper on the "Stetho-Sphygmograph," as an aid to the physiological and pathological investigation of the functions of respiration and circulation. He exhibited the practical application of the instrument on a case of phthisis pulmonalis, and one of well-marked mitral regurgitation. In every case three simultaneous and synchronous tracings of moving organs are taken—as, for example, the two lungs and the radial pulse; or the heart, the radial pulse, the carotid, or the femoral. It was shown that the tracings would show any disparity in the action of the two lungs, also the relation of inspiration to expiration; and, associated with the pulse tracing, it afforded the opportunity of observing, not only the peculiarities or modifications of the circulation, as in the case of Marey's sphygmograph, but in addition, their relation to the respiratory process. By aid of this instrument, any question as to relative time in the transmission of the blood-wave through the arteries may be solved; as, for example, the time occupied in transmission to the radial, the carotid, or the femoral artery. This may be found to have important connection with the diagnosis of aneurisms and tumors, as well as with diseased states of the arteries with chronic diseases of organs.—*Lond. Lancet.*

---

CHLOROFORM IN THE TREATMENT OF BILIARY CALCULI. By JOHN BARCLAY, M.D., Physician to the Infirmary, Leicester.—Seeing some reference in a contemporary periodical to the proposed use of chloral as a solvent of biliary calculi, I crave space to state that I have met with very great success from the internal administration of chloroform in that disease.

I first used it in 1861, in the case of a clergyman, aged 58. He had suffered for twenty-three years from gall-stones; the peculiar pain, jaundice, with subsequent discharge, by stool, of the calculi, coming on so suddenly and without warning, as seriously and frequently to interfere with his duties. Just then, writing on alcohol, I had been studying the experiments of Lallemand and others on the existence of alcohol unchanged in the blood. Knowing that ethers are solvents of cholesterine, I ventured, on his third attack in that year, to prescribe chloroform in doses of two or three drops, three or four times a day, on the chance of its reaching the calculi through the blood. To his surprise and my gratification, pain, tenderness, distention and jaundice disappeared together, and in the eight years since elapsed, he has never had another attack. He keeps a bottle of "chloric ether" by him, for occasional use. I have found it to give invariable and permanent relief in many instances since.

The theory of thus dissolving the calculi *in situ*, followed by the disappearance of the symptoms, leads to a deduction that may be legitimate, that relief was obtained by their being so dissolved.—*British Medical Journal.*

---

A New Vesicant.—In a recent number of the *Journal de Chimie Medicale*, M. Delpech calls attention to the defects of the ordinary preparations of cantharides; the proportion of the active agent, or cantharidin, varying, the presence of fatty or oily substances sometimes causing the absorption of a dangerous poison, and the rosin or turpentine employed in the composition of the plaster being an unnecessary irritant. In place of this, he recommends the cantharidinate of potash, which is of a very stable composition, has no odor, and possesses a vesicant action in a high degree. He recommends as the best formula for the plaster:—Gelatin, 2.09 parts; water, 10 parts; alcohol, 10 parts; cantharidinate of potash, 0.20 part; glycerine, 9.5 parts. The mass should be equally spread on thin gutta percha, a definite quantity being present in each square inch.—*Lancet.*

---

The Value of American Hemp.—Dr. H. C. Wood, Jr. has written an essay which he read before the Amer. Phil. Society, in which he records some experiments with an article of hemp grown in Kentucky. He took an alcoholic extract made from the dried leaves, swallowing at a dose nearly all the product of an ounce and a-half of the leaves, with the effect of profound hemp intoxication. It proved to be toxic in its power, although he recovered himself in a day or two. He had all the exuberant hilarity usually experienced from the hemp, followed by a feeling of fear of impending death; this took so deep a hold on him that it was impossible to shake it off.

Other trials he has made with it convince him that it has more power than that brought from India, on one occasion four times the dose of the latter failing to produce the effect of the Kentucky specimen.

He has his extract made from a tincture, removing certain inert matters by an alkali; he intimates the hope that in the present revision of the U. S. Pharmacopœia the *ex cannibis purifactum* may be replaced by a preparation to be called *Resena cannibis*, and to be made by precipitating the concentrated tincture by water rendered strongly alkaline by soda of potash.

The native plant if used will always be more reliable than the imported, from the certainty of freshness, while the cost of it is hardly anything.

### Mortality for the Month of August, 1870.

| | |
|---|---|
| Accidents, by chloroform | 1 |
| " solution of potash | 1 |
| " shooting | 1 |
| " by team | 1 |
| " drowned | 11 |
| " by fall | 3 |
| " railroad | 4 |
| Anasarca | 1 |
| Apthæ | 2 |
| Apoplexy | 1 |
| Abscesses scrofulous | 1 |
| Anæmia | 1 |
| Aortic valves, ossification of and hyperthrophy of heart | 1 |
| Births, premature | 7 |
| Births still | 46 |
| Bowels, ulceration of | 1 |
| Brain, congestion | 10 |
| " inflammation | 18 |
| " effusion on | 1 |
| " softening | 1 |
| " and hemiplegia | 1 |
| Bronchitis | 8 |
| " capillary | 1 |
| Cancer of liver | 1 |
| " orbit | 1 |
| " pylorus | 1 |
| " rectum | 1 |
| " tongue | 1 |
| " stomach | 3 |
| " " uterus | 2 |
| Cholera Infantum | 277 |
| " morbus | 6 |
| Consumption | 44 |
| Convulsions | 82 |
| Croup | 10 |
| " membranous | 3 |
| Cynanche tonsillaris | 2 |
| Development deficient abdomem | 1 |
| Debility, general | 9 |
| Delirium Tremens | 1 |

| | |
|---|---|
| Diabetes Mellitus | 1 |
| Diarrhœa | 55 |
| " chronic | 10 |
| " & complications | 11 |
| Diphtheria | 17 |
| Dropsy | 2 |
| Dysentery | 40 |
| " & paralysis | 1 |
| " acute | 1 |
| " chronic | 4 |
| " typhoid | 6 |
| Entero-colitis | 6 |
| Enteritis | 15 |
| Exhaustion following ligation of artery | 1 |
| Fever, congestive | 3 |
| " puerperal, | 5 |
| " remittent | 4 |
| " scarlet | 16 |
| " " complications | 3 |
| " " malignant | 2 |
| " typhoid | 38 |
| " typhus | 2 |
| Gastro-Enteritis | 6 |
| Heart, disease of & albuminuria | 1 |
| " dropsy of | 1 |
| " valvular disease | 4 |
| Hepatitis, chronic | 2 |
| Hip disease | 1 |
| Hydrocephalus | 23 |
| " acute | 6 |
| " chronic | 1 |
| Hydro-peritonium | 1 |
| Inanition | 25 |
| Intemperance | 2 |
| Icterus | 1 |
| Kidneys, Bright's disease | 2 |
| " organic disease of & dropsy | 1 |
| " & liver tuberculosis | 1 |
| Laryngitis | 1 |

| | |
|---|---|
| Liver, organic disease & dropsy of | 1 |
| Lungs, congestion | 5 |
| Measles | 2 |
| " & complications | 2 |
| Metro peritonits | 1 |
| " & puerperal | 3 |
| Meningitis | 7 |
| " & erysipelas | 1 |
| " cerebro-spinal | 4 |
| " tubercular | 5 |
| Meritis & overitis | 1 |
| Old age | 11 |
| " and dropsy | 1 |
| Paralysis | 4 |
| Peritonitis | 6 |
| Pneumonia | 11 |
| " bronche | 1 |
| " typhoid | 2 |
| Pyæmia | 2 |
| Scrofula | 1 |
| Small-Pox | 1 |
| Spine, paralysis | 1 |
| Stomatitis | 1 |
| Stomach, traumatic inflammation | 1 |
| Suicide by arsenic | 1 |
| " " laudan'm | 1 |
| " " morphine | 1 |
| " " shooting | 1 |
| Tabes mesenterica | 51 |
| Teething | 26 |
| " & complications | 20 |
| Throat, malig't sore | 1 |
| Trismus | 2 |
| Uterus, inflammation | 1 |
| Urethra, stricture | 1 |
| " rupture of & extravagasation of urine | 1 |
| Whooping-cough | 14 |
| Total | 1033 |

### COMPARISON.

Deaths in Aug., 1870, 1033 | Deaths in Aug. 1869, 1072 | Decrease, 39
Deaths in July, 1870, 1120 | Decrease, 87

### AGES.

| | | | | | |
|---|---|---|---|---|---|
| Under 1 | 471 | 10 to 20 | 30 | 70 to 80 | 15 |
| 1 to 2 | 223 | 20 to 30 | 75 | 80 to 90 | 3 |
| 2 to 3 | 44 | 30 to 40 | 46 | Unknown, | 1 |
| 3 to 4 | 22 | 40 to 50 | 40 | | |
| 4 to 5 | 9 | 50 to 60 | 24 | Total, | 1033 |
| 5 to 10 | 21 | 60 to 70 | 9 | | |

| | | |
|---|---|---|
| Males, ............547 | Females, ............486 | Total, ........1033 |
| Single, ............885 | Married ............148 | Total, ........1033 |
| White, ............1022 | Colored, ............11 | Total, ........1033 |

NATIVITY.

| | | | | | |
|---|---|---|---|---|---|
| Atlantic Ocean | 1 | England | 20 | Nova Scotia | 1 |
| Bohemia | 9 | France | 1 | Switzerland | 1 |
| Canada | 2 | Germany | 75 | Scotland, | 7 |
| Chicago, Native | 144 | Holland | 4 | Sweden | 7 |
| Chicago, Foreign | 577 | Ireland | 62 | Unknown | 4 |
| U. S., other parts | 99 | Isle of Jersey | 1 | | |
| Denmark | 1 | Norway | 17 | Total, | 1033 |

MORTALITY BY WARDS FOR THE MONTH.

| Wards. | Mortality. | Wards. | Mortality. |
|---|---|---|---|
| 1 | 11 | 18 | 70 |
| 2 | 20 | 19 | 17 |
| 3 | 36 | 20 | 23 |
| 4 | 18 | Accidents | 22 |
| 5 | 35 | County Hospital | 15 |
| 6 | 92 | Convent Good Shepherd | 1 |
| 7 | 81 | Home for Friendless | 5 |
| 8 | 91 | Hospital Alexian Brothers | 6 |
| 9 | 82 | Immigrants | 17 |
| 10 | 17 | Mercy Hospital | 7 |
| 11 | 34 | Erring Woman's Refuge, | 1 |
| 12 | 35 | Protestant Orphan Asylum | 3 |
| 13 | 20 | St. Joseph Orphan Asylum | 10 |
| 14 | 25 | Suicide | 4 |
| 15 | 98 | | |
| 16 | 62 | Total, | 1033 |
| 17 | 75 | | |

### A GOOD CHANCE FOR A GOOD PHYSICIAN.

I wish to dispose of my house and two lots in the village of Two Rivers, with a good village and country practice. Said lots are conveniently and pleasantly located, and have a good house, barn, and other improvements. For particulars and reasons for wishing to sell, address,

C. C. CROCKER, M.D.,
Two Rivers, Manitowoc County, Wis.

